# Evidence-based summary of optimal home self-management for elderly ostomy patients

**DOI:** 10.3389/fmed.2026.1807235

**Published:** 2026-06-01

**Authors:** Jiayi Feng, Fang Lu, Bo Zhou, Ji Li, Jian Shen, Lulu Tang, Yangyang Chen, Liping Kang, Guanghui Li, Zhaobei Wang, Huiqin Lin, Yuehua Gao

**Affiliations:** Infectious Surgery Department, Guiyang Public Health Clinical Center, Guiyang, Guizhou, China

**Keywords:** aged, enterostomy, evidence-based nursing, home care, self-management

## Abstract

**Background:**

Given the aging population and increasing incidence of severe intestinal diseases, elderly ostomy patients face home self-management challenges with insufficient care capacity, and the lack of systematically integrated evidence for their specific needs necessitates summarizing the best evidence to support clinical care and improve their quality of life.

**Methods:**

A computerized literature search was systematically performed to identify studies focusing on home stoma self-management among elderly ostomy patients. The search was conducted across multiple sources, including domestic and international guideline platforms, official websites of relevant institutions, and major academic databases. Eligible literature included clinical practice guidelines, clinical decision-making tools, expert consensus statements, systematic reviews, and evidence summaries. Literature was searched from January 2015 to October 2025 in the following resources.

**Results:**

A total of 15 high-quality studies were included, encompassing six core domains: self-management goals, stoma assessment, psychological adjustment, dietary management, behavioral management and medication management.

**Conclusion:**

A total of 36 pieces of evidence were summarized in this study, which fills the gap of systematically integrated evidence for home stoma self-management in elderly ostomy patients. However, given that this evidence was derived from diverse countries with diverse healthcare systems and contexts, its clinical application should be tailored to the specific clinical conditions and individual needs of this population. Future research should focus on developing more targeted, evidence-based interventions to optimize home stoma self-management for elderly ostomy patients.

**Reporting method:**

This evidence summary followed the reporting specifications for evidence summaries developed by the Fudan University Center for Evidence-based Nursing, which were based on the methodological framework for evidence summaries produced by the Joanna Briggs Institute (JBI). The reporting process included establishing the evidence-based question, literature retrieval, literature screening, quality appraisal, evidence synthesis and grading, and development of practice recommendations. This study was registered in the evidence summary registration system of Fudan University Center for Evidence-based nursing under the title “Evidence-based summary of optimal home self-management for elderly ostomy patients” with the registration number ES20246036.

**Patient or public participation:**

No direct on-site patient or public participants were recruited for this literature-based evidence summary. All patient-related experiences and practical perspectives were extracted and integrated from the included published studies to ensure the applicability of the optimal home self-management evidence for elderly ostomy patients.

**Systematic review registration:**

Identifier ES20246036.

## 1 Introduction

To date, colorectal cancer has become one of the top three most prevalent diseases globally, with approximately 1.5 million new cases diagnosed each year. Among these patients, 20% to 30% require ostomy surgery (1). Data indicates that approximately 102,000 individuals in the United States have an ostomy, with about 21,000 new cases occurring annually. In the United Kingdom, approximately 100,000 new colostomy patients and 10,000 new ileostomy patients are recorded each year (2, 3). China has nearly 100,000 new ostomy patients each year, with the cumulative number of patients exceeding one million and an annual growth rate of 4.2% (4). Although the surgery has significantly improved the patient’s five-year survival rate to over 60%, it also brought about a series of adverse effects on the patient’s physical health, daily life, and even psychological well-being (5).

Additionally, elderly ostomy patients at home face challenges such as poor self-care capabilities and low levels of psychological and social adaptation, which increase the readmission risk and disease burden, severely impacting recovery and quality of life (6). Especially for elderly ostomy patients, the lack of professional support from healthcare providers at home presents significant challenges in self-management of their stoma (7). In severe cases, this may even lead to anxiety or depression (8).

Currently, research on home self-management measures for ostomy patients remains relatively scarce worldwide. Existing literature primarily focuses on specific aspects, such as bowel habit guidance for ostomy patients and evaluations of health education outcomes. Moreover, most studies focus only on specific stages, with little attention paid to summarizing the main problems encountered in the home-based phase. Consequently, the associated adverse problems have not been effectively addressed.

Therefore, this study focuses on home self-stoma management among elderly ostomy patients. It systematically retrieves relevant domestic and international literature on home self-stoma management for elderly ostomy patients, subsequently screening and synthesizing scientific evidence. To provide scientific evidence for standardized home self-stoma management among elderly ostomy patients.

## 2 Methods/Methodology

### 2.1 Problem establishment

The PIPOST developed by the Evidence-Based Nursing Center (9) establish an evidence-based problem framewor. (1) P (Population): Target population: Elderly patients with intestinal ostomies. (2) I (Intervention): Intervention methods: Including treatment measures, assessment methods, and intervention strategies for elderly patients with intestinal ostomies. (3) P (Professional): Evidence users: Clinical practitioners. (4) O (Outcome) Outcome Measures: Incidence of complications, quality of life, and readmission rates among elderly ostomy patients. (5) S (Setting) Evidence Application Settings: Surgical wards and patients’ homes. (6) T (Type of Evidence) Evidence Types: guidelines, expert consensus statements, clinical decision aids, systematic reviews, and evidence summaries.

### 2.2 Inclusion and exclusion criteria

The inclusion criteria were as follows: (1) study subjects: elderly patients with enterostomy; (2) study types: clinical guidelines, systematic reviews, evidence-based summaries, randomized controlled trials, and cohort studies; (3) core content: home self-management, stoma care, complication prevention, diet, exercise, psychosocial adaptation, or family support; (4) language: Chinese or English.

Studies were excluded if they focused on perioperative management only, were expert opinions without evidence grading, were animal experiments, case reports, or studies with incomplete data.

### 2.3 Literature search strategy

Based on the “6S” evidence mode (10) systematically search domestic and international guideline networks, databases, and relevant institutional websites. A comprehensive systematic search was conducted in electronic databases including PubMed, Web of Science, Embase, Cochrane Library, CNKI, Wanfang, and CBM. The search terms combined medical subject headings and free words: aged, elderly, ostomy, enterostomy, stoma, self-management, home care, self-care. Boolean operators: (Aged OR Elderly) AND (Ostomy OR Enterostomy OR Stoma) AND (Self-management OR Home care). The search period was from January 2015 to October 2025.

### 2.4 Literature screening

The obtained literatures were imported into EndNote and duplicates were deleted. Two researchers trained in evidence-based medicine independently screened the literatures. The title, abstract and keywords were read during preliminary screening. Next, the full texts were read and rescreened, and the quality of the rescreened literatures were evaluated. During the evaluation if the inclusion of the obtained literature was controversial, the literatures were discussed with a third critical field evidence-based care specialist to determine inclusion status.

### 2.5 Quality evaluation of the literature

Different assessment tools were used according to study types. Clinical practice guidelines: AGREE II, Systematic reviews: AMSTAR 2, Randomized controlled trials: JBI RCT critical appraisal tool, Cross-sectional/cohort studies: JBI observational study critical appraisal tool (3, 11, 12). The Up To Date clinical decision and JBI summary of evidence are in the upper part of the evidence resources pyramid, thus, the level and quality of evidence were high. Meanwhile, no corresponding tool exists to evaluate the quality of evidence summaries and clinical decision. Consequently, the quality of these types of evidence was judged by tracing the original document of each evidence source and selecting the corresponding evaluation tool for quality evaluation. Quality scores and evaluation results were recorded and summarised for each included study.

The present study used the AGREE II that was updated in 2017 and the scoring criteria included 23 items across six fields, including scope, purpose, participants, etc. On the AGREE II scale, a score of 1 corresponds to strongly disagree and 7 to strongly agree for the evaluation of each item. Standardized scores in each field = (actual score evaluated−lowest possible score)/(highest possible score − lowest possible score) × 100%. The guidelines recommend division of the standardization score into three grades: if 6 items score ≥60%, it is recommended as a grade A; if ≥3 items score ≥30% with some items with scores <60%, it is recommended as a grade B after modification and improvement; and ≥ 3 areas with scores <30% are excluded as grade C.

The systematic evaluation tool of the Assessment of Multiple Systematic Reviews 2 (AMSTAR 2) was used to evaluate systematic reviews in this study. The evaluation method includes 16 entries, of which 7 are key entries. Literature with no or only 1 non-key entry nonconformity is considered high quality; literature with 1 or more than 1 non-key entry nonconformity is considered medium quality;

The Expert Consensus Evaluation Criteria of JBI Evidence-Based Health Care Centerswas adopted to evaluate expert consensus in this study. The evaluation tool includes six items, including the source of opinions, the author’s influence in the field, whether the views are centred on relevant stakeholders, the logic of the views, whether to refer to other literature and whether the view is consistent with the previous. The evaluation results of each item are judged by “yes,” “no,” “unclear” and “not applicable.” Cohort studies were evaluated by the evaluation criteria of the JBI Evidence-Based Health Care Centre in Australia.

The team consists of three researchers trained in systematic evidence-based methods. Two researchers evaluate studies based on quality assessment criteria and determine eligibility for inclusion. In cases of disagreement, the three researchers discuss the findings, with the third researcher making the final decision. When evidence types conflict, priority is given to guidelines, expert consensus, high-quality evidence, and the most recent evidence.

### 2.6 Evidences extraction and summary

Synthesize the evidence ultimately included. Based on the JBI Evidence Pre-rating and Recommendation Level System (2014 Edition) (13), we conducted quality assessments and assigned recommendation levels to the included evidence. The grading criteria categorize the included evidence into levels 1 to 5, with level 1 being the highest and level 5 the lowest.

The feasibility, appropriateness, meaningfulness and effectiveness evaluation principle was used to evaluate the evidence. In addition, expert meetings were held to demonstrate the recommended strength of the evidence, and finally, the recommendation strength was divided into strong recommendation (grade A) and weak recommendation (grade B) based on the JBI recommendation grading.

## 3 Results

### 3.1 Search results

**Figure 1:**
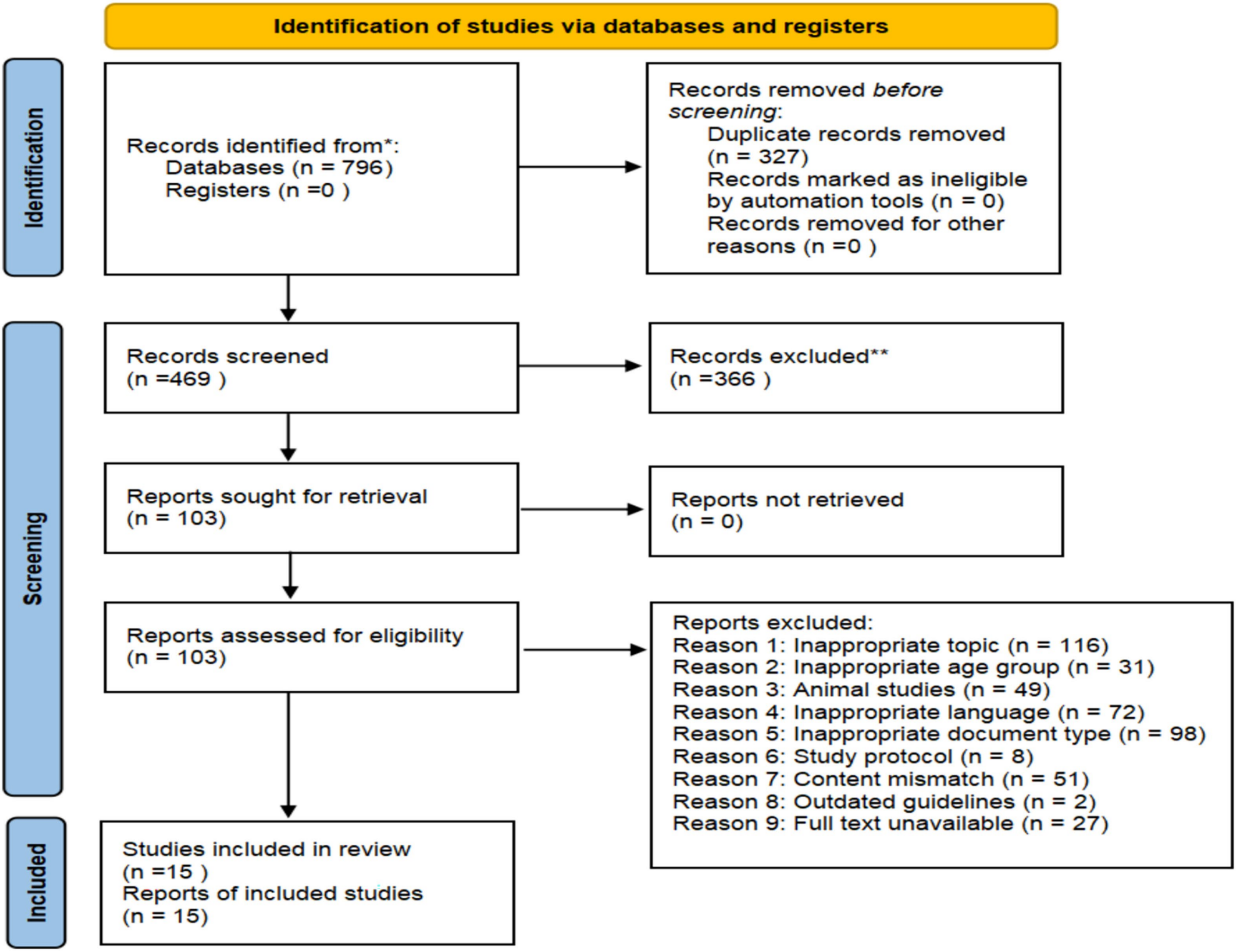
PRISMA 2020 flow diagram of literature screening.

A total of 796 records were retrieved from databases and other sources. After removing 327 duplicates, 469 records were screened by title and abstract, and 103 full-text articles were assessed for eligibility. Following full-text evaluation and quality appraisal, 15 studies were finally included. The literature screening process is presented in Figure 1 (PRISMA 2020 flow diagram). The general characteristics of the included studies are summarised in Table 1.

**Table 1:** General characteristics of the included literatures (*n* = 15).

Included literature	Year of publication (year)	Literature reference	The literature theme	Type of literature
Lightner et al. (19)	2022	YIMAITONG	Geriatric ostomy care management	Guideline
Chabal et al. (16)	2021	WCET	Ostomy care	Guideline
Díaz-Pizarro et al. (17)	2020	PubMed	Nutritional support for elderly patients with intestinal fistula	Guideline
Ferrara et al. (18)	2019	PubMed	Therapeutic measures for geriatric ostomy	Guideline
Wound, Ostomy and Continence Nurses Society; Guideline Development Task Force (15)	2018	WOCN	Ostomy patient care	Guideline
Miller et al. (20)	2017	ETN	Promoting postoperative care for ostomy patients	Guideline
Stelton et al. (21)	2014	WCET	International stoma care	Guideline
The Committee for Wound Ostomy Incontinence Care. Standards for Adult Ostomy Care (42)	2022	CNKI	Adult colostomy care standards	Expert consensus
Colwell et al. (22)	2019	Ovid	Assessment of peristomal skin and follow-up management	Expert consensus
Landmann and Cashman (14)	2021	Up To Date	Management of colostomy complications	Clinical decision
Wang et al. (3)	2022	CNKI	Comprehensive colostomy care management	Summary of evidence
Si et al. (41)	2021	CNKI	Perioperative health education for colostomy patients	Summary of evidence
Goodman et al. (24)	2022	PubMed	Self-management for stoma patients	Systematic review
Jin et al. (26)	2022	PubMed	Impact of continuous care on stoma patient health	Systematic review
Capilla-Díaz et al. (25)	2019	PubMed	Living with a stoma: a qualitative systematic review	Systematic review

### 3.2 Quality evaluation results of the included literature

#### 3.2.1 Clinical decisions and summary of evidence

As shown in Table 2, included 1 clinical decision (14) and 2 evidence summaries (3), all quality evaluations are rated A-level recommendations and approved for inclusion.

**Table 2:** Clinical decision-making and evidence synthesis quality assessment results (*n* = 3).

Items	Included in the literature		
	Landmann and Cashman (14)	Wang et al. (3)	Si et al. (41)
1. Is the scope and application specific?	Yes	Yes	Yes
2. Are the authors transparent?	Yes	Yes	Yes
3. Is the review process clear and transparent?	Yes	Yes	Yes
4. Is the search method transparent and comprehensive?	Yes	Yes	Yes
5. Is the evidence classification clear?	Yes	Yes	Yes
6. Is the recommendation clear?	Yes	Yes	Yes
7. Have these suggestions been properly cited?	Yes	Yes	Yes
8. Is the recommendation cutting-edge?	Yes	Yes	Yes
9. Has potential bias been avoided?	Yes	Yes	Yes
10. Is the target audience clearly defined?	Yes	Yes	Yes

#### 3.2.2 Quality evaluation results of guidelines

As shown in Table 3, a total of 7 guidelines were included (15–21), two researchers carefully evaluated each guideline across six criteria: scope and purpose, participants, rigor of development, clarity, applicability, and editorial independence. Ultimately, 4 documents were assigned a recommendation level of Grade A, and 3 documents were assigned a recommendation level of Grade B.

**Table 3:** Quality evaluation results of clinical guidelines (*n* = 7).

Guideline	Standardized scores in various domains (%)						≥60%	≥30%	Quality evaluation
	Scope and purpose	Stakeholder involvement	Rigour of development	Clarity of presentation	Applicability	Editorial independence			
Lightner et al. (19)	96	72	83	94	69	100	6	6	A
Chabal et al. (16)	100	48	59	93	67	100	4	6	B
Díaz-Pizarro et al. (17)	100	68	67	88	72	83	6	6	A
Ferrara et al. (18)	87	70	74	91	61	92	6	6	A
Wound, Ostomy and Continence Nurses Society; Guideline Development Task Force (15)	93	74	78	87	65	100	6	6	A
Miller et al. (20)	70	77	64	42	83	67	5	6	B
Stelton et al. (21)	83	42	79	81	67	50	5	6	B

#### 3.2.3 Quality evaluation results of expert consensus

As shown in Table 4, included 2 expert consensus documents, evaluated using the JBI Literature Quality Assessment Criteria (2016 edition), literature (22) except for item 6, which is “unclear,” all other items are evaluated as “Yes”; Literature (23) all six entries are “Yes.” Two expert consensus documents were included due to their overall high quality and well-designed research methodologies.

**Table 4:** Quality evaluation results of expert consensus (*n* = 2).

Items	The Committee for Wound Ostomy Incontinence Care. Standards for Adult Ostomy Care (42)	Colwell et al. (22)
1. Is the source of the opinion clearly identified?	Yes	Yes
2. Does the source of opinion have standing in the field of expertise?	Yes	Yes
3. Are the interests of the relevant population the central focus ofthe opinion?	Yes	Yes
4. Is the stated position the resultof an analytical process, and is there logic in the opinion expressed?	Yes	Yes
5. Is there reference to the extant literature?	Yes	Yes
6. Is any incongruence with the literature/sources logically defended?	Yes	Unclear

#### 3.2.4 Quality evaluation results of systematic reviews

As shown in Table 5, Included in 3 systematic reviews, literature (24) except for Item 6, which is “Unclear,” all other items are evaluated as “Yes”; 2 remaining documents (25, 26) all 11 evaluation results were “Yes.” Three studies demonstrated high overall quality with detailed research designs and were included.

**Table 5:** Quality evaluation results of systematic review (*n* = 3).

Items	Included literature		
	Goodman et al. (24)	Jin et al. (26)	Capilla-Díaz et al. (25)
1. Is the evidence-based question clearly defined?	Yes	Yes	Yes
2. Are the inclusion criteria for the literature appropriate?	Yes	Yes	Yes
3. Is the search strategy appropriate?	Yes	Yes	Yes
4. Are the sources of research papers appropriate?	Yes	Yes	Yes
5. Are the quality criteria for the literature appropriate?	Yes	Yes	Yes
6. Was the literature quality assessment performed independently by two or more evaluators?	Unclear	Yes	Yes
7. Were measures taken to minimize errors during data extraction?	Yes	Yes	Yes
8. Was the method for synthesizing/pooling studies accurate?	Yes	Yes	Yes
9. Was potential publication bias assessed?	Yes	Yes	Yes
10. Were recommendations for policy and/or practice supported by reported data?	Yes	Yes	Yes
11. Were appropriate suggestions made for specific directions of future research?	Yes	Yes	Yes

### 3.3 Summary and description of evidence

As shown in Table 6, this study conducted a comprehensive literature search, quality assessment, and evidence grading on home self-stoma management for elderly ostomy patients. It extracted high-level evidence regarding patient goals, stoma assessment, psychological adjustment, dietary management, behavioral management, medication management, and self-monitoring in home self-stoma care, ultimately compiling 36 best evidence statements.

**Table 6:** Evidence summary of home self-management for elderly ostomy patients.

Category	Content of evidence	Level	Recommendation level
Self-management goals	1. Elderly ostomy patients can master ostomy-related knowledge and self-care skills required for ostomy management at home (24).	5	A
Stoma assessment	2. Monitor and replace the ostomy pouch appropriately, preferably on an empty stomach in the early morning (18).	5	A
	3. Replace the pouch promptly if leakage, itching, or burning sensation occurs around the stoma (22).	5	B
	4. Initiate immediate pouch replacement upon signs of leakage, itching, or burning around the stoma (16).	5	B
	5. Regularly assess the stoma and peristomal skin to maintain skin health (14, 22).	5	A
	6. Accurately measure abdominal dimensions and select an appropriate abdominal belt; the belt length should cover the stoma and pouch (18).	5	B
	7. Choose suitable ostomy care products; use a soft pouch when a parastomal hernia is present (22).	5	B
	8. Patients should learn to recognize common symptoms and potential complications, such as stoma ischemia, bleeding, necrosis, retraction, skin-mucosal separation, parastomal hernia, prolapse, and stricture, to facilitate early prevention of stoma-related pathologies (14, 18).	5	B
Psychological adjustment	9. Recommend that family members or caregivers directly participate in stoma care to provide psychological support to patients (41).	5	A
	10. Stoma surgery patients should proactively seek stoma care experience from fellow stoma patients to enhance confidence in self-management (21, 41).	5	B
	11. Encourage stoma patients experiencing anxiety, depression, or grief to actively seek help from psychologists, stoma nurses, or other professionals (24).	5	B
	12. Patients are advised to prepare psychologically before surgery for life with a temporary or permanent stoma, including adapting to new bowel habits and changes in self-image (25).	1	B
Dietary management	13. Educate patients to recognize symptoms of delayed gastric emptying and abdominal distension (Chinese Nursing Association, 2022).	5	B
	14. Recommend daily protein intake of 1.5–2.0 g/kg, high-output stomas require 2.5 g/kg (3, 17).	5	B
	15. Maintain a BMI of 20–25 kg/m^2^ to help reduce the risk of parastomal hernia (20, 22).	5	B
	16. Colostomy patients should maintain adequate fiber (20–35 g/day) and fluid intake (≥1.5–2 L/day) to prevent constipation; (3, 17, 24).	5	B
	17. Enteral or parenteral nutrition is recommended for low- and high-output stoma patients to meet fluid, electrolyte, and nutritional requirements (3, 17).	5	B
	18. Daily monitoring of fluid intake and output is advised. Avoid consuming indigestible or easily clumping foods, and limit intake of gas-producing, odorous, spicy, or raw/cold foods (4, 14).	1	B
Behavioral management	19. Provide a simple stoma care manual and supply checklist for home use (24, 41).	5	B
	20. Train patients to perform self-emptying and pouch changes using demonstration-based education (41).	5	B
	21. Ensure secure pouch attachment during outings; avoid extreme sports (21).	5	B
	22. Avoid gas-producing activities like carbonated beverages and chewing gum ((20); Chinese Nursing Association, 2022).	1	B
	23. Recommend that patients undergoing ostomy surgery participate in online and offline learning programs related to ostomy issues. (41).	5	A
	24. Master pouch placement, emptying, and replacement techniques and timing; select appropriate ostomy supplies (16).	5	B
	25. During exercise or sweating, ensure secure pouch adhesion and maintain pouch integrity (16).	5	B
	26. Avoid direct skin contact with abdominal belts to prevent pressure-related skin issues (22).	5	A
	27. Perform weekly anal dilation to prevent stoma stenosis (14, 18)	5	B
	28. Resume sexual activity after physical recovery to minimize stoma-related impact (25).	1	B
	29. Initiate gentle abdominal exercises within 3 months after surgery and maintain for at least 1 year (18).	5	B
	30. Apply a convex baseplate and ostomy belt in cases of stoma retraction (16).	5	B
	31. Use barrier creams, strips, rings, or hydrocolloid dressings to maintain peristomal skin integrity and prevent fecal contamination (22).	5	B
Drug management	32. Advise patients to consult a stoma nurse before using medications with unclear indications (22).	5	B
	33. For peristomal skin–mucosal separation, refer to a physician for appropriate antibiotic or wound treatment (18).	5	B
	34. Avoid using medications that are poorly soluble or absorbable, such as controlled-release and enteric-coated formulations (14).	5	B
	35. When patients experience stoma pain, immediate use of analgesics is not recommended to prevent masking of underlying conditions (26).	1	B
	36. Instruct patients not to adjust drug dosage or timing independently; follow medical advice based on stoma type and output (16, 41).	5	B

## 4 Discussion

### 4.1 Self-management goals

Self-management ability is a key indicator for assessing a patient’s stoma recovery and quality of life. The World Health Organization (WHO) notes that self-management plays a crucial role in chronic disease care (27). Based on existing literature, the self-care and management abilities of ostomy patients in China generally remain at a low to moderate level (28). Therefore, guidelines recommend that patients and their families actively participate in stoma care during hospitalization to enhance patients’ ability to self-manage their stoma at home (29). The home self-management goal for elderly ostomy patients in this study is to master ostomy-related knowledge and essential stoma care skills (15, 18, 20, 25). However, most of the included evidence supporting self-management goal setting is level 5 evidence (primarily expert consensus and evidence summaries), which offers practical clinical guidance but has relatively low methodological strength. Thus, the goal of enabling elderly patients to master basic ostomy knowledge and self-care skills should be considered clinically meaningful but not strongly evidence-based. Clinical application should be combined with individualized health education, and future high-level intervention studies are needed to verify whether achieving such goals can effectively improve long-term outcomes.

### 4.2 Stoma assessment

Accurate assessment of the skin condition around the stoma and timely ostomy pouch changes are key measures for effectively reducing adverse reactions in home ostomy patients. Research indicates that ostomy patients typically experience increased stool and gas output upon waking. Therefore, guidelines recommend emptying the ostomy pouch in the early morning on an empty stomach or replacing it when waste fills one-third to one-half of the pouch’s total capacity. This practice effectively prevents discomfort caused by delayed pouch changes (19). Especially when the stoma base appears white, curled, or when skin itching, burning, or leakage occurs, the ostomy pouch should be changed (3). Guidebook (42) recommended to use a “stool diary” to assess the volume, color, and consistency of the patient’s ostomy output, facilitating prompt medical attention should abnormalities arise. It is worth noting that patients with parastomal hernias should opt for soft ostomy pouches to prevent skin breakdown (26). However, this type of gusseted bag has been subject to some controversy due to its tendency to sag under weight and its reduced adhesive effectiveness. Additionally, experts recommend that the length of the abdominal binder should cover the stoma and ostomy pouch to maintain the health of the skin surrounding the stoma (Chinese Nursing Association, 2022). However, for key links such as the selection of ostomy products and the use of abdominal belts, since the evidence is mostly low-level and context-dependent, clinical application should fully consider the patient’s economic status, physical condition, and family support. Data indicates that without effective home management, ostomy patients may experience ostomy retraction, skin-mucosal separation, paraostomy hernia, prolapse, and stricture (3). Therefore, the guidelines recommend that elderly ostomy patients learn to recognize common symptoms and potential complications to enable early prevention of ostomy-related conditions (18). However, elderly ostomy patients exhibit significant variations in educational attainment, making it challenging for them to recognize ostomy-related adverse conditions, although these suggestions are widely used in clinical practice, their supporting evidence is indirect and lacks large-sample prospective verification.

### 4.3 Psychological adjustment

Psychological adjustment is a crucial aspect of self-management for elderly ostomy patients living at home. Research indicates that individuals with stomas not only face challenges in stoma management and care, but also must adapt to the physiological and psychological changes brought about by the stoma creation process (30). In particular, lack of support or understanding from family members of ostomy patients can significantly impact the patient’s psychological well-being and, to some extent, increase the cost of ostomy care (31). While existing literature supports that psychological interventions may contribute to improved adaptation outcomes, most recommendations are based on consensus and clinical experience rather than large-scale quantitative data. Additionally, due to the unique nature of their condition, ostomy patients exhibit a higher incidence of anxiety and depression compared to non-ostomy patients (32). Guide (Chinese Nursing Association, 2022) recommend that elderly ostomy patients prepare mentally in advance for adapting to life with a temporary or permanent stoma, including adjusting to new bowel habits and changes in self-image. Encourage ostomy patients to actively seek ostomy care advice from fellow ostomates, build confidence in self-management, monitor their own emotional responses, and proactively seek assistance from mental health professionals, ostomy nurses, and other support resources (31). However, current self-regulation strategies generally lack consistency and practicality, and their effectiveness may vary depending on individual differences and cultural backgrounds.

### 4.4 Dietary management

Dietary management is a crucial component of self-care for elderly ostomy patients living at home. Research indicates that poor nutrition significantly impacts patients’ risks of wound healing, pressure ulcer development, and readmission (33). Available guidelines (34) suggest that ostomy patients may monitor daily fluid intake and output, avoid foods that are difficult to digest or likely to clump, and limit intake of gas-producing, odorous, spicy, or raw/cold items. Research indicates that while surgical procedures remain the primary treatment for elderly ostomy patients, dietary and nutritional requirements vary depending on the specific location of each patient’s surgical stoma (35). Low protein levels have been associated with higher risks of postoperative complications in elderly ostomy patients. Accordingly, some guidelines (36) suggest a daily protein intake of 1.5–2.0 g/kg to help meet disease-related nutritional needs. Cao et al. (37) and Huang et al. (38) indicate that malnutrition is an effective predictor of complications in ostomy patients, and malnourished patients exhibit poorer self-reported health outcomes. Guidelines also recommend enteral or parenteral nutrition for elderly ostomy patients with both low and high output to meet fluid, electrolyte, and nutritional requirements. Specifically, 20–35 g/day of fiber can prevent constipation (Chinese Nursing Association, 2022). Additionally, adequate carbohydrate intake can meet the energy requirements for metabolic changes caused by stomas and wound healing in patients (39). In summary, maintaining adequate nutritional intake is crucial for the prognosis and recovery of ostomy patients. Most of the evidence supporting dietary recommendations is derived from guidelines and consensus-based sources, with relatively few high-level quantitative studies. The overall quality of the current evidence base is variable, indicating a need for future targeted studies to develop more site-specific and validated dietary management protocols for elderly ostomy patients. Such evidence would help support more individualized and clinically informed dietary choices for this population.

### 4.5 Behavior management

Behavioral interventions represent commonly used and clinically practical approaches to support independent self-management among elderly ostomy patients in the home setting. Guidelines (16) suggest that elderly ostomy patients learn ostomy-related information through both online and offline formats, particularly focusing on methods for emptying and changing ostomy pouches. Self-management capacity is regarded as a relevant indicator for monitoring stoma recovery and reducing complication risks. Therefore, it may be beneficial for patients to develop a simple stoma self-care manual and supply checklist to support consistent self-management (17). Nonetheless, such tools may need adaptation to accommodate age-related and educational limitations commonly seen in the target population. In addition, to minimize the impact of stoma on patients’ sexual activity, experts suggest gradually resuming sexual activity after physical recovery (25). Research indicates that smoking is a risk factor for developing para-stoma hernias, with an incidence rate four times higher than in non-smokers. Therefore, it is recommended to avoid gas-producing behaviors such as drinking carbonated beverages and chewing gum (22). Ostomy patients may be advised to wear loose-fitting clothing to prevent pressure on the stoma. When away from home, attention should be paid to secure pouch attachment. Strenuous or high-impact physical activities are generally not recommended for safety. During physical activity or periods of heavy sweating, it is prudent to monitor pouch adhesion and seal integrity (29). Research indicates that weekly anal dilation can effectively prevent stoma stenosis in elderly ostomy patients (24). It is also recommended to begin abdominal exercises within 3 months after stoma creation and continue them for at least 1 year (20). In cases of stoma retraction, clinical experience and available guidelines support use of a convex baseplate secured with a stoma belt to reduce risks of fecal contamination (3). However, it is noteworthy that at present, elderly ostomy patients primarily rely on video tutorials and health guidance manuals as behavioral management tools. Yet these resources are often provided by ostomy product companies, whose content emphasizes product promotion rather than fostering patient engagement and long-term self-management. Consequently, they contribute little to enhancing ostomy patient’s self-management awareness and capabilities.

### 4.6 Drug management

Self-medication management methods for elderly ostomy patients at home are primarily divided into oral and topical categories. Some evidence indicate that without timely drug prophylaxis and intervention, severe adverse reactions may develop over time, with a 1-year complication rate at home ranging from 33% to 44% (40). Based on clinical consensus, early application of stoma powder may help reduce peristomal skin damage (22). For mild erythema and moderate to heavy exudate on stoma skin, absorbent dressings such as polyurethane foam, sodium alginate, or hydrofiber may be considered (18). In cases of unclear medication use or severe skin damage, consult a stoma nurse for specific medication instructions. Research indicates that due to the diminished intestinal absorption capacity of elderly ostomy patients, medications that are difficult to dissolve or absorb-such as controlled-release and enteric-coated formulations-should be avoided (21). Research indicates that one-third of elderly ostomates experience stoma pain. However, consensus-based guidance advises caution with immediate analgesic use, to help avoid masking potential underlying conditions (3). At present, high-level evidence supporting home medication management regimens for elderly ostomy patients remains limited globally. Since medication needs vary by stoma site, it is reasonable that patients should manage their medications under medical supervision, according to stoma type and effluent volume.

### 4.7 Limitations

Although this study comprehensively summarized the evidence on home self-management of intestinal stomas in elderly patients, regional, racial, and cultural differences may have influenced the findings. Furthermore, only English and Chinese databases were searched; literature published in other languages was not included. The evidence base requires ongoing updating in future research, and applicable evidence should be selected according to feasibility, suitability, effectiveness, and clinical context to facilitate practical implementation.

## 5 Conclusion

This study summarizes the best available evidence regarding home self-stoma management for elderly ostomy patients. The 36 summarized evidence-based guidelines are recommended primarily for clinical reference. Considering the substantial individual variability among older adults and regional bias arising from the predominance of overseas literature, these findings should be applied cautiously and individualized according to a comprehensive assessment of each patient’s clinical status and personal needs. Domestic high-quality research remains limited; further rigorous studies are warranted to enrich the current evidence base and facilitate improvements in patients’ quality of life.

## 6 Relevance to clinical practice

Home self-management of ostomies remains a persistent challenge for elderly ostomy patients. This study synthesizes 36 evidence-based recommendations to optimize home self-care among elderly ostomy patients. Appropriate adoption and implementation of these 36 evidence points may facilitate standardized self-management practices for elderly ostomy patients in clinical settings.

These 36 pieces of evidence cover six core domains: self-management goals, stoma assessment, psychological adjustment, dietary management, behavioral management, and medication management. Collectively, these six domains cover the major dimensions of home-based self-stoma care.

Stoma assessment is integral throughout the entire intervention process and serves as a critical foundation for developing individualized self-management strategies for elderly ostomy patients.

This study provides a valuable reference for healthcare professionals worldwide engaged in home-based self-stoma management for elderly ostomy patients.

## Data Availability

The raw data supporting the conclusions of this article will be made available by the authors, without undue reservation.
